# Saponin Micelles Lead to High Mucosal Permeation and In Vivo Efficacy of Solubilized Budesonide

**DOI:** 10.3390/pharmaceutics12090847

**Published:** 2020-09-05

**Authors:** Sabine Nakowitsch, Christiane Koller, Jan-Marcus Seifert, Marielle König-Schuster, Nicole Unger-Manhart, Cornelia Siegl, Norman Kirchoff, Elisabeth Foglar, Christine Graf, Martina Morokutti-Kurz, Marianne Neurath, Svenja Sladek, Christian Knecht, Wolfgang Sipos, Eva Prieschl-Grassauer, Andreas Grassauer

**Affiliations:** 1 Marinomed Biotech AG, Hovengasse 25, 2100 Korneuburg, Austria; christiane.koller@marinomed.com (C.K.); jan-marcus.seifert@marinomed.com (J.-M.S.); marielle.koenig-schuster@marinomed.com (M.K.-S.); nicole.unger-manhart@marinomed.com (N.U.-M.); cornelia.siegl@marinomed.com (C.S.); KirchoffN@rki.de (N.K.); elisabeth.foglar@gmx.at (E.F.); christine.graf2@chello.at (C.G.); martina.morokutti-kurz@marinomed.com (M.M.-K.); marianne.neurath@marinomed.com (M.N.); svenja.sladek@marinomed.com (S.S.); eva.prieschl@marinomed.com (E.P.-G.); andreas.grassauer@marinomed.com (A.G.); 2 Clinical Department for Farm Animals and Herd Management, University of Veterinary Medicine Vienna, Veterinärplatz 1, 1210 Vienna, Austria; christian.knecht@vetmeduni.ac.at (C.K.); Wolfgang.Sipos@vetmeduni.ac.at (W.S.)

**Keywords:** solubilization, micelles, inflammation mouse model, drug permeation, local application, tissue availability

## Abstract

Due to fast nasal mucociliary clearance, only the dissolved drug content can effectively permeate the mucosa and be pharmaceutically active after intranasal application of suspensions. Therefore, the aim of this study was to increase the budesonide concentration in solution of a nasal spray formulation. Budesonide, a highly water-insoluble corticosteroid, was successfully solubilized using a micellar formulation comprising escin, propylene glycol and dexpanthenol in an aqueous buffered environment (“Budesolv”). A formulation based on this micellar system was well-tolerated in the nasal cavity as shown in a good laboratory practice (GLP) local tolerance study in rabbits. Ex vivo permeation studies into porcine nasal mucosa revealed a faster and more efficient absorption. Budesolv with 300 µg/mL solubilized budesonide resulted in a budesonide concentration of 42 µg/g tissue after only 15 min incubation. In comparison, incubation with the marketed product Rhinocort^®^ aqua 64 (1.28 mg/mL budesonide as suspension) led to 15 µg/g tissue. The in vivo tumor-necrosis-factor (TNF)-α secretion in an acute lung inflammation mouse model was significantly reduced (*p* < 0.001) following a prophylactic treatment with Budesolv compared to Rhinocort^®^ aqua 64. Successful treatment 15 min after the challenge was only possible with Budesolv (40% reduction of TNF-α, *p* = 0.0012) suggesting a faster onset of action. The data reveal that solubilization based on saponin micelles presents an opportunity for the development of products containing hardly soluble substances that result in a faster onset and a better topical treatment effect.

## 1. Introduction

The rate and extent of drug absorption into a tissue after topical application determines the bioavailability and the effectivity of a locally acting drug. The main determinants for this drug influx are drug dissolution, drug permeability properties and applied drug concentration [1]. Despite the importance of drug dissolution, about 40% of approved drugs and nearly 90% of the developmental pipeline drugs represent highly permeable, but poorly water soluble molecules [2].

In order to achieve higher bioavailability, academia and industry have been and still are continuously putting a lot of effort into increasing drug solubility, for example by particle size reduction (micronization), crystal engineering, pH adjustment, salt formation, solid dispersion, co-solvency, the use of surfactants or complexation [3,4].

In this study, we sought to develop an efficient and well-tolerated system to solubilize budesonide, a highly effective anti-inflammatory glucocorticoid, often applied as nasal spray for treatment of allergic rhinitis. Due to its hydrophobic nature, budesonide shows a limited degree of solvation (solubility of 28 µg/mL) [5,6], but high tissue permeability. Therefore, budesonide-containing nasal sprays, such as the marketed Rhinocort^®^ aqua 64 (AstraZeneca, Vienna, Austria), are usually formulated as an aqueous suspension with only a small fraction of the active pharmaceutical ingredient in solution (less than 3%). After intranasal application of a drug, effective permeation needs to occur within 20–30 min before the compound is transported into the pharynx due to mucociliary clearance [7,8]. However, for drug permeation into the nasal mucosa, the micronized, solid particles of the suspension first have to dissolve in the nasal fluid [9]. Therefore, approximately 50–70% of intranasally applied budesonide from conventional nasal sprays is swallowed [3], not available at the target site and subsequently inactivated through the first pass effect [4].

In the past, other attempts have been made to solubilize budesonide for intranasal application. One of them was the complexation with cyclodextrins (CDs); the resulting formulations were also tested preclinically [10] and clinically [11]. As the clinical trials with the budesonide CD formulation did not result in increased efficacy in comparison to the suspension, one aim of this study was to compare tissue availability and anti-inflammatory efficacy of budesonide solubilized with the presented system with those achievable with CD-solubilized budesonide.

CDs are a well-established system for solubilization of lipophilic and amphiphilic substances [12]. They are able to form water soluble complexes with lipophilic drugs in their core, which are reported to be released at a controlled [3] or slow rate [13]. Local toxicity and the influence on the ciliary beat frequency after nasal administration are very low [14]. This makes CDs advantageous over the use of organic solvents for solubility enhancement [15].

One of the oldest and simplest methods to increase the solubility of poorly water-soluble molecules is the addition of surfactants. Surfactants are amphiphilic molecules that contain both hydrophobic and hydrophilic groups. When the concentration of surfactants in an aqueous environment exceeds a certain threshold, the surfactant and formulation specific critical micelle concentration (CMC) micelles are formed, which can incorporate a hydrophobic drug into the micellar structure. The polar hydrophilic groups of the surfactant molecules face outwards, hereby forming a hydrophilic outer layer in contact with the surrounding aqueous environment. The hydrophobic part of the surfactant faces inwards creating a hydrophobic core [4].

The ability of some naturally occurring saponins to form micelles has been known for many years. Saponins are used in the food industry to enhance the solubility and/or dispersion stability of incompatible mixtures [16,17]. Saponins comprise a hydrophobic triterpenoid or steroidal aglycone structure, also called sapogenin, with one or more hydrophilic glycoside moieties attached. Naturally occurring saponins differ in the type and subcategory of their aglycone structures. as well as the number, type and position of the attached glycoside moieties. Monodesmosidic saponins have only one sugar chain, usually attached to the C-3 of the aglycone, while bidesmosides have an additional glycosylation at C-28 of the aglycone [17].

The well-known saponin escin, which is a mixture of 30 different related molecular species extracted from horse chestnut (*Aesculus hippocastanum*), mainly contains monodesmosides with a trisaccharide attached at the C-3 of the aglycone [18,19,20]. Escin has long been used for the treatment of various conditions including chronic venous insufficiency and has an excellent safety profile [21]. Orally. it is administered at very high doses of up to 150 mg/day, while topically applied gels contain ≥1% escin. Escin was already investigated for the solubilization of the hydrophobic drugs fenofibrate and danazol [22], where it showed a strong solubilization effect only for the steroidal structured danazol. We previously published the use of escin as part of our solubilization platform Marinosolv^®^ for the development of tacrolimus-containing eye drops, which showed increased tissue concentrations of permeated tacrolimus in different eye compartments in a porcine ex vivo and in vivo eye model compared to a tacrolimus suspension [23].

Marinosolv^®^ represents a proprietary platform of formulations that utilizes various saponins, a low concentration of pharmaceutically or cosmetically acceptable co-solvents and dexpanthenol as a general principle to solubilize hydrophobic, highly insoluble molecules, including active pharmaceutical ingredients.

Here we describe the development of an aqueous nasal formulation of solubilized budesonide (from here on called “Budesolv”) based on the Marinosolv^®^ platform. For the solubilization of budesonide, the saponin escin and dexpanthenol, as well as propylene glycol (PG) as co-solvent, were used. Dexpanthenol, also called d-panthenol, is the hydroxy analog of d-pantothenic acid. Dexpanthenol is used in a wide variety of products, e.g., in food (it is “generally recognized as safe”, (GRAS) status since 1978 with an estimated mean intake of 7 mg/day), in cosmetics, medical devices and medicinal products [24]. Dexpanthenol is frequently used in intranasal formulations in concentrations of up to 5% (*w*/*v*) [25]. As a medium-chain polyol, dexpanthenol has the potential to act as a co-solvent and/or co-surfactant in aqueous micellar formulations. Alcohols have long been known as co-surfactants in microemulsions that can potentially further enhance the solubility of water-insoluble compounds [26]. Micelle formation in aqueous solutions can reportedly be favored by the addition of polyols to the formulation [27]. The underlying mechanism was found to be an elongation of the micelle shape caused by a dehydration of the hydrophilic part of the amphiphile. In the present work we analyzed the drug permeation in ex vivo experiments. The anti-inflammatory activity of budesonide applied as Budesolv (300 µg/mL) was characterized in comparison to CD-solubilized budesonide (300 µg/mL) and the commercial product Rhinocort^®^ aqua 64 micrograms, nasal spray (originally marketed by AstraZeneca; 1.28 mg/mL) in in vivo experiments. Additionally, we proved the local tolerance of intranasally applied Budesolv in a four-week GLP study in rabbits.

## 2. Materials and Methods

### 2.1. Budesonide Solubilization

To prepare the different formulations, 0–0.05% *w*/*v* escin (Euro OTC, Bönen, Germany) was dissolved in phosphate/citrate buffer at pH 6, consisting of 5.88 mg/mL Na_2_HPO_4_·2H_2_O (MERCK, Darmstadt, Germany), 1.78 mg/mL citric acid monohydrate (MERCK, Darmstadt, Germany) and 1 mg/mL Titriplex III (MERCK, Darmstadt Germany). Budesonide (Crystal Pharma, Boecillo, Spain) was pre-dissolved in propylene glycol (PG; Sigma-Aldrich, St. Louis, MO, USA) and added to the different formulations under stirring at room temperature to a final concentration of 10% *v*/*v* PG or a maximal concentration of 1100 µg/mL budesonide. The influence of dexpanthenol on the solubilization of budesonide was investigated by preparing the above described solutions, including 0–0.05% escin, with 6% *w*/*v* dexpanthenol (Fluka, Waltham, MA, USA) before addition of the budesonide stock solution. As control samples, dry micronized budesonide (without pre-solvation in PG) was added to aqueous buffered solutions containing 0–0.05% *w*/*v* escin with and without dexpanthenol under continuous stirring. Determination of budesonide concentration was done by Reversed Phase-High Performance Liquid Chromatography (RP-HPLC). Samples were centrifuged (10 min at 15,800 rcf) to sediment undissolved particles. Clear supernatants were transferred and used to determine the solubilized budesonide content by a calibrated RP-HPLC. Every step of the procedure before the HPLC analysis was done at room-temperature. Analysis was performed at isocratic conditions (55% acetonitrile (Chemlab, Zedelgem, Belgium)/45% water with 0.01% TFA (MERCK, Darmstadt, Germany); 1 mL/min flow rate) using a Thermo Fisher Dionex Ultimate 3000 HPLC system with an Agilent Zorbax SB-C18 (3.5 µm 4.6 × 150 mm) column and UV–Vis detection at 244 nm. The RP-HPLC was calibrated using ten different calibration samples in the range of 0.5–16 µg budesonide control reference standard (The European Directorate for the Quality of Medicines & HealthCare [EDQM], Cat. No. B1157300, batch 4.1) with triplicate measurements. The triplicate analysis of the calibration samples showed a maximal standard deviation of 0.48% and the according calibration curve had a linearity of *R*^2^ = 0.9999. The determined limit of detection was 0.14 µg budesonide control reference standard and the limit of quantification was 0.43 µg budesonide control reference standard. Formulation samples were routinely analyzed in duplicates and results were considered valid with a relative standard deviation of <1%.

### 2.2. Determination of the Critical Micelle Concentration (CMC) of Escin

Escin was dissolved in concentrations from 0.008% to 1.0% *w*/*v* in a phosphate/citrate buffer at pH 6 before addition of PG to a final concentration of 5% *v*/*v*. Samples were mixed with 2.5 µL of a 1.4 mM stock solution of the fluorescence dye bisBenzimidine H33342 (Sigma Aldrich, St. Louis, MO, USA) yielding a final concentration of 7 µM. After preparation, 100 µL of each sample were transferred to a black HTS-Microplates 96-well plate with µClear^®^ bottom (Greiner, Kremsmünster, Austria) (in duplicates). As described by Jumpertz et al. [28] an increase of fluorescence emission occurs upon incorporation of the dye into the hydrophobic core of a micelle. H33342 fluorescence was measured with a Fluostar Omega microplate reader (BMG LABTECH Gmbh, Ortenberg, Germany) set to 355 nm and 460 nm for excitation and emission, respectively. The CMC was calculated by applying a sigmoidal fit to the respective data sets (xlfit, version 5.5.0.5, IDBS, Guildford, UK, 2017). The tangent at the inflection point was determined. The x-coordinate of the intersection of the tangent with a parallel line to the *x*-axis through the lowest measured value represents the CMC.

### 2.3. Ex Vivo Permeation Experiments on Porcine Nasal Mucosa

Budesolv formulations were prepared as described above. Escin (0.03% *w*/*v*) was dissolved in a phosphate/citrate buffer at pH 6 and different concentrations of budesonide (25 µg/mL, 180 µg/mL, 200 µg/mL, 300 µg/mL, 500 µg/mL, 620 µg/mL and 800 µg/mL) as PG stock solution were added under stirring. The final solutions contained 10% PG. The β-CD-containing formulation was prepared by dissolving 2.86 mg/mL randomly methylated β-cyclodextrins (CAVASOL W7 M, Wacker, Munich, Germany) in 0.9% NaCl (ready to use solution) and then adding 300 µg/mL budesonide under stirring. Rhinocort^®^ aqua 64 micrograms nasal spray (AstraZeneca, Vienna, Austria, Ch.B. S1114A) was used.

Fresh porcine nasal mucosa was obtained from pigs euthanized in the course of other experiments or for diagnostic reasons, but with no history of nasal discharge or upper airway insufficiency, at the University Clinic for Swine, University of Veterinary Medicine Vienna. Uniform parts of the nasal mucosa were taken with a 10 mm biopsy punch (78 mm^2^ area) and stored in Ringer’s solution at room temperature for ≤1 h. Prior to treatment, liquid was drained off and the nasal mucosa tissue pieces were weighed (between 60–120 mg/piece). Nasal mucosa pieces were placed apical side up into a 48-well cell culture plate and 50 µL/100 mg tissue of the respective formulation was applied onto the mucosa surface. Samples were incubated for up to 60 min in a humidity chamber. After the respective incubation period, treated mucosa pieces were rinsed 5 times with ringer solution, frozen in liquid nitrogen, and stored at −80 °C until analysis. Quantification of permeated budesonide was done by mass spectrometry HPLC-MS/MS after homogenization of the tissue pieces with a Precellys 24 homogenizer (Peqlab, Erlangen, Germany). For homogenization per 100 mg tissue, 700 µL 50% methanol (Honeywell, Morristown, NJ, USA) was added and homogenization was done with 6 cycles of 20 s at maximum speed with cooling intervals. To 25 µL of this homogenate, 300 ng/mL Diazepam (Sigma-Aldrich, St. Louis, MO, USA) as internal standard was added and samples were protein precipitated with 50 µL acetonitrile (MERCK, Darmstadt, Germany). After centrifugation for 10 min at room temperature (8000 rpm in an Eppendorf 5417C centrifuge), the supernatants were diluted with water (1 + 1, *v*/*v*) and transferred to a brown glass vial for HPLC-MS/MS analysis. Samples were analyzed at following gradient HPLC conditions: A: water with 0.1% formic acid (Sigma-Aldrich, St. Louis, MO, USA) and B: acetonitrile with 0.1% formic acid organic phase; % B (t (min)), 5% (0–0.1) – 97% (0.6–1.7) – 5% (1.8–2.5); 600 µL/min flow rate using a Kinetex Phenyl-Hexyl column, 2.6 µm, 50 × 2 mm (Phenomenex, Aschaffenburg, Germany) with a Security Guard precolumn (Phenomenex, Aschaffenburg, Germany). HPLC-MS/MS analysis was performed by Pharmacelsus GmbH (Saarbruecken, Germany) with a Triple Quadrupole mass spectrometer (TSQ Quantum Discovery Max, Thermo Fisher, Waltham, MA, USA) with ESI (positive electrospray ionization mode).

### 2.4. Acute Murine Lung Inflammation Experiment

Animal experiments were approved by the institutional ethics committee and performed under the Austrian experimental license numbers BMWF-68.205/0163-WF/V/3b/2015 and BMWFW-68.205/0175-WF/V/3b/2017. All animal experiments were carried out in accordance with Directive 2010/63/EU for animal experiments. Female BALB/c mice (8 weeks old) were purchased from JANVIER Labs (France) and housed at standard conditions with a 12-h light/dark cycle. Water and pelleted food (complete diet for rodents from Ssniff/Germany) were provided ad libitum. Mice were anesthetized with Vetflurane (Virbac, Vienna, Austria) and treated by pipetting 50 µL of the respective treatment solution into each nostril in an upright position. Treatment was done in five mice per group with the following formulations: 0.9% NaCl as placebo (ready to use solution for human use, Braun, Germany); Budesolv, containing 300 µg budesonide solubilized in 0.03% *w*/*v* escin in a phosphate/citrate buffer and 5% *v*/*v* PG; Rhinocort^®^ aqua 64 micrograms nasal spray (AstraZeneca), containing 1.28 mg/mL dispersed budesonide; β-CD: 300 µg/mL budesonide solubilized in 2.86 mg/mL randomly methylated β-cyclodextrins in 0.9% NaCl (ready to use solution).

Treatment was done 24 h, 18 h and 3 h before or 15 min after acute lung inflammation was induced by administration of 3 mg/kg body weight *E. coli* lipopolysaccharide (LPS) O55:B5 (Sigma-Aldrich, St. Louis, MO, USA) under anesthesia in the same way as described for the treatment. Two hours after LPS challenge mice were sacrificed and lungs were flushed with 1 mL ice-cold PBS to obtain bronchoalveolar lavage (BAL). BAL was stored at −80°C until analysis. TNF-α concentration was assessed with the commercially available mouse TNF-α ELISA MAX™ Deluxe Set (BioLegend, San Diego, CA, USA).

TNF-α values of different treatment groups were calculated for their statistical significance using one-way ANOVA followed by post hoc analysis using the Bonferroni–Holm correction. For all evaluations, values of *p* < 0.05 were assessed significant.

### 2.5. Four-Week Intranasal Local Tolerance and Toxicity GLP Studies in Rabbits

A 28-day intranasal GLP safety and tolerance study in NZW rabbits was conducted at LPT Laboratory of Pharmacology and Toxicology GmbH & Co. KG, Hamburg, Germany, according CPMP/SWP/1042/99 Rev1, to test the local tolerance of Budesolv. Following formulations were tested: Budesolv 300 µg/mL; Budesolv 150 µg/mL; Rhinocort^®^ aqua 64 micrograms nasal spray (AstraZeneca). Budesolv formulations were prepared in a phosphate/citrate buffer at pH 4.5 with addition of 0.3 mg/mL escin, 2.5 mg/mL NaCl (Fluka, Waltham, MA, USA), 0.4 mg/mL ascorbic acid (Sigma-Aldrich, St. Louis, MO, USA) and 60 mg/mL dexpanthenol. Final Budesolv formulations contained 10% *v*/*v* PG with either 300 µg/mL or 150 µg/mL budesonide.

Budesolv 300 µg/mL and Budesolv 150 µg/mL were applied twice daily, 100 µL each into one nostril. In the respective other nostril 100 µL vehicle (matched placebo formulations containing all excipients without budesonide) were applied twice daily. Rhinocort^®^ aqua 64 micrograms nasal spray was applied twice daily, 50 µL into one nostril.

## 3. Results

### 3.1. Investigation of Solubilizing Agents for Budesonide

**Figure 1:**
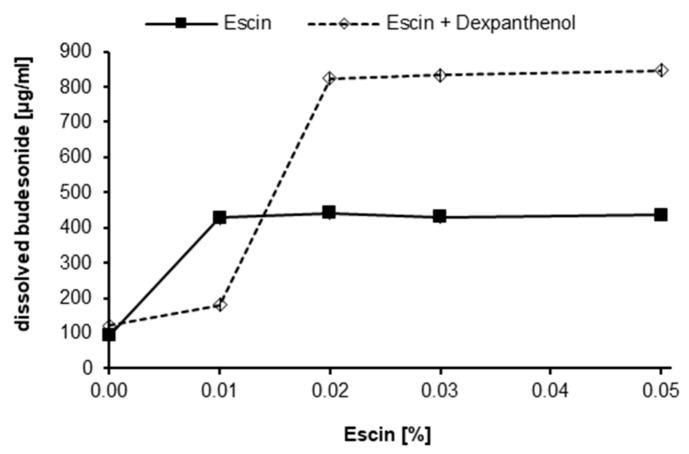
Effect of escin and dexpanthenol on the solubilization of budesonide. Concentrations of budesonide (µg/mL, *x*-axis) determined by RP-HPLC in formulations containing 0–0.05% escin (*y*-axis) with and without 6% dexpanthenol measured after 6 days stored at room temperature. Data are expressed as mean (*n* = 2).

Initially, we determined the solubility of budesonide in aqueous formulations in the absence and presence of the saponin escin. Budesonide was dissolved in PG and subsequently mixed with buffered solutions to yield final formulations comprising 10% PG and 0–0.05% escin. Six days after preparation, the concentration of solubilized budesonide was determined by RP-HPLC (Figure 1). In the aqueous buffer control without escin and PG, the solubility of budesonide was found in four independent experiments to be on average 28.9 µg/mL. In the presence of 0.01% escin with 10% PG, the solubilized content of budesonide was 419 µg/mL. Increasing escin concentrations above 0.01% did not result in a substantial increase of solubilized budesonide. The experiment revealed that the addition of escin and 10% PG enhances the concentration of budesonide in solution by a factor of approximately 10 in comparison to the buffer control. In control samples, where budesonide was added as micronized powder, no increased solubilization of budesonide was found. A formulation containing 0.05% escin and 6% dexpanthenol, yielded 33.71 µg/mL of budesonide in solution.

Furthermore, by incorporating 6% dexpanthenol to escin containing solutions prior to the addition of the budesonide stock solution, >800 µg/mL budesonide were solubilized (Figure 1). In an additional long-term experiment, solubilized budesonide in formulations containing 5% PG without dexpanthenol tended to precipitate after several weeks. Specifically, without dexpanthenol only 41% of the initially solubilized budesonide were still in solution after 3 months, whereas in a corresponding formulation with 6% dexpanthenol, 92% of the initially solubilized budesonide concentration was found (data not shown). These results suggest that dexpanthenol might act as a co-solubilizing agent but might also improve the long-term colloidal stability of the micellar formulation.

### 3.2. Characterization of Micellar Formulation

The CMC of saponins is not a specific material constant, but influenced by factors such as pH, temperature and salt concentration of the formulation [16,20]. Therefore, the CMC of escin in our buffered formulation was measured using the fluorescent probe bisBenzimide H33342, which shows an increase of fluorescence emission upon incorporation into the hydrophobic core of the micelle [28]. In this assay, the CMC for escin in our formulation system was found to be 0.01%. Furthermore, the addition of dexpanthenol did not have an influence on the CMC (data not shown).

To this point, characterization of size, size distribution and morphology of the escin micelles in Marinosolv^®^ using dynamic light scattering (DLS) have been unsuccessful. This is most likely due to the low concentration of micelles in the formulation, their potentially non-spherical shape, and a heterogenous size distribution. Transmission electron microscopy (TEM) imaging has also been unsuccessful. The difference in refractive index between surrounding media and micelles might not be sufficient. Furthermore, the preparation procedure for TEM imaging could have negative effects on the micelle integrity.

### 3.3. Ex Vivo Permeation Kinetics Experiments on Porcine Nasal Mucosa

**Figure 2:**
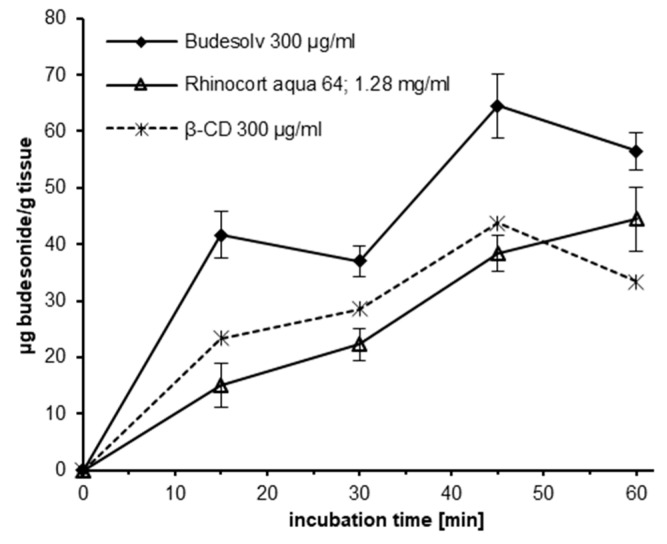
Permeation of budesonide into porcine nasal mucosa. Budesonide tissue concentrations (µg/g tissue as measured by HPLC-MS/MS, *y*-axis) in porcine nasal mucosa treated with either Budesolv or β-CD complex containing 300 µg/mL budesonide or Rhinocort^®^ aqua 64 containing 1.28 mg/mL budesonide, were evaluated after different incubation time points (*x*-axis). Data are expressed as mean ± standard deviations (SD) (*n* = 3).

The ex vivo permeation of 300 µg/mL solubilized budesonide into excised porcine nasal mucosa from Budesolv and β-CD complexes was compared to the permeation of budesonide from Rhinocort^®^ aqua 64, containing 1.28 mg/mL budesonide in suspension (Figure 2). Freshly excised porcine nasal mucosa was incubated with the different formulations, and the amount of budesonide permeated over time was quantified by HPLC-MS/MS. Statistical analysis of the permeated amount of budesonide into the mucosal tissue from different formulations at different time points was done with paired Student’s *t*-test and is shown in Table 1. Generally, it can be stated that at all time points a significantly higher amount of budesonide from Budesolv compared to β-CD complex with the same concentration of solubilized budesonide or the marketed product with a significantly higher concentration of dispersed budesonide permeated into the mucosa. In contrast, the tissue concentration of the samples incubated with 300 µg/mL solubilized budesonide as β-CD complex compared to Rhinocort^®^ aqua 64 was only significantly higher at the 30 min time point.

**Figure 3:**
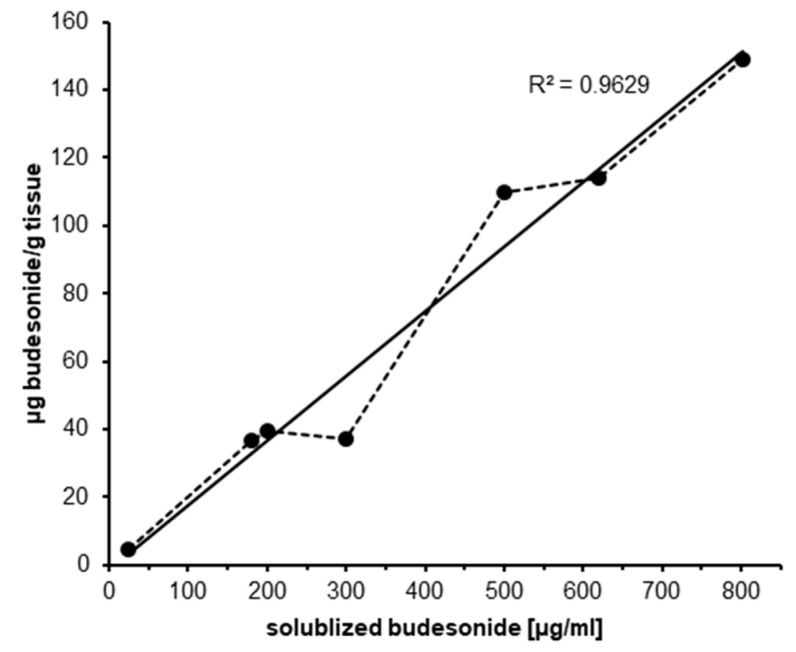
Relationship of permeated vs. applied concentration of solubilized budesonide. The amount of permeated budesonide (µg/g tissue, *y*-axis) measured in porcine mucosa samples (expressed as mean of *n* = 3–5) after 30 min of incubation with Budesolv formulations containing different concentrations of solubilized budesonide (µg/mL, *x*-axis). The correlation coefficient *R*^2^ was calculated by linear regression, for which the trend line is displayed. The data from several individual experiments were used to generate this general relationship.

In further experiments the permeation of budesonide from Budesolv formulations with different concentrations of solubilized budesonide was investigated as described above (Figure 3). The amount of budesonide permeated after 30 min of incubation was quantified by HPLC-MS/MS. A clear correlation of the amount of permeated budesonide and the applied concentration of solubilized budesonide was found (*R*^2^ = 0.9629).

In conclusion, these experiments showed that drug solubilization by micelles results in a faster uptake and a higher total drug tissue concentration in the mucosa. In addition, the experiments showed that the nasal mucosa can absorb a very high amount of budesonide if there is no restriction in time of uptake or dissolved drug content.

### 3.4. In Vivo Investigation of the Anti-Inflammatory Efficacy in Mice

The efficacy of formulations containing solubilized budesonide and budesonide in suspension was compared in an acute lung inflammation mouse model to determine whether the higher tissue levels and faster uptake observed ex vivo correlate with a better efficacy of solubilized budesonide. Animals were treated intranasally with Budesolv (300 µg/mL budesonide), Rhinocort^®^ aqua 64 (1.28 mg/mL dispersed budesonide) or placebo. At different time points (24 h, 18 h and 3 h) after the respective treatment, mice were challenged with LPS. Two hours after the LPS challenge, the bronchoalveolar lavage (BAL) of the mice was obtained for TNF-α quantification.

**Figure 4:**
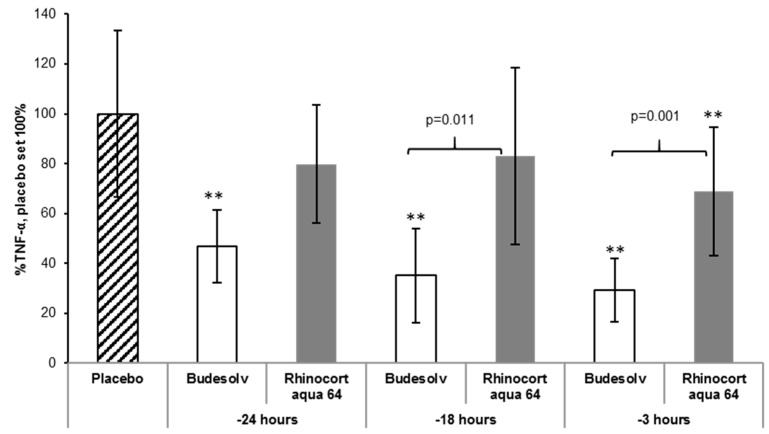
In vivo TNF-α quantification in a prophylactic set-up. Determined concentration of TNF-α (*y*-axis, normalized to Placebo) in murine BAL after intranasal treatment with either Budesolv 300 µg/mL, Rhinocort^®^ aqua 64 (1.28 mg/mL dispersed budesonide) or Placebo (*x*-axis). LPS challenge was applied 24 h, 18 h, or 3 h after treatment (*x*-axis). Data are expressed as mean ± SD (*n* = 5). Statistical analysis was done by One-way ANOVA with Bonferroni’s post-test. ** represent *p* < 0.001 in comparison to placebo.

As shown in Figure 4, the treatment with Budesolv 300 µg/mL led to a significant reduction of TNF-α in comparison to placebo at all time points (*p* < 0.001). In contrast, Rhinocort^®^ aqua 64 showed a significant reduction of TNF-α in comparison to placebo only at one time point (treatment 3 h before LPS challenge). At earlier time points, Rhinocort^®^ aqua 64 did not significantly reduce the inflammatory reaction to the LPS challenge, as measured by TNF-α in the BAL fluid, in comparison to placebo. In addition, the treatment with Budesolv 300 µg/mL significantly reduced the TNF-α secretion in comparison to Rhinocort^®^ aqua 64 at 18 h and 3 h before LPS challenge.

The fast permeation of budesonide in the ex vivo experiments suggested a potentially faster onset of anti-inflammatory action. To test this hypothesis, a second in vivo experiment with a therapeutic set-up (treatment after the LPS challenge) instead of the prophylactic application of budesonide (treatment before the LPS challenge) was performed. Mice were first challenged with LPS and treated with the respective formulations after 15 min. As described above, TNF-α was quantified in the BAL fluid two hours post LPS challenge. In this set up, additionally to Budesolv (300 µg/mL budesonide) and Rhinocort^®^ aqua 64 (1.28 mg/mL dispersed budesonide), budesonide solubilized with β-CD complexes (300 µg/mL budesonide) was tested.

**Figure 5:**
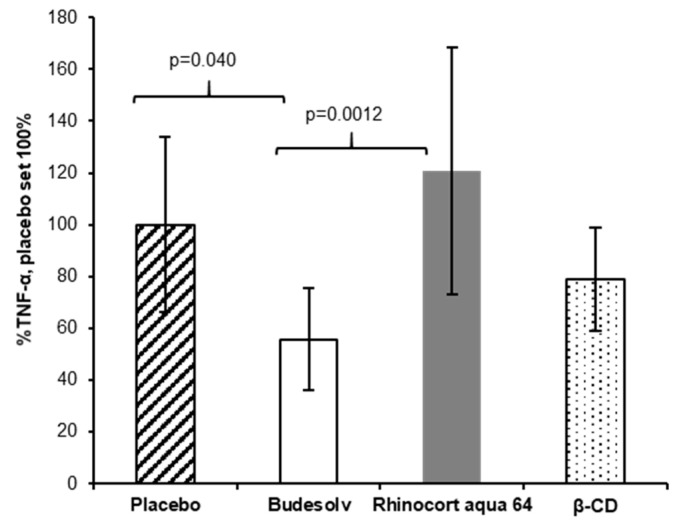
In vivo TNF-α quantification in a therapeutic set-up. Determined concentrations of TNF-α (*y*-axis, normalized to Placebo) in murine BAL after intranasal treatment with either Budesolv 300 µg/mL, Rhinocort^®^ aqua 64 (1.28 mg/mL dispersed budesonide), a β-CD formulation containing 300 µg/mL solubilized budesonide, or Placebo (*x*-axis) done 15 min after LPS challenge. Data are expressed as mean ± SD (*n* = 5). Statistical analysis was done by One-way ANOVA with Bonferroni’s post-test.

As shown in Figure 5, the treatments with Rhinocort^®^ aqua 64 and the budesonide β-CD complexes did not result in a statistically significant reduction of the TNF-α response. On the other hand, the treatment with Budesolv was already effective in inhibiting TNF-α secretion (*p* = 0.040 versus placebo) 15 min post LPS challenge. Again, when comparing Budesolv to Rhinocort^®^ aqua 64, Budesolv resulted in a significantly reduced TNF-α secretion (*p* = 0.0012). The budesonide β-CD complex (300 µg/mL budesonide) showed a trend for a better therapeutic in vivo activity compared to Rhinocort^®^ aqua 64 (not significant). These results are in accordance to Dufour et al., who previously showed that a 2.5× lower concentrated solution of budesonide HP-β-CD complexes yields similar activity in a mouse ovalbumin induced challenge model compared to a budesonide suspension [10].

Overall, these results show that the faster permeation of solubilized budesonide observed ex vivo translate into a faster onset of action and subsequently a better therapeutic effect in vivo. This introduces the opportunity for a more effective treatment of an already initiated inflammation.

### 3.5. Four-Week Intranasal Local Tolerance and Toxicity GLP Study in Rabbits

Safety of a formulation is an important parameter. Therefore, a GLP study was conducted to obtain information on the intranasal safety and tolerance of Budesolv in rabbits after twice daily intranasal administration over a period of 28 days. The study was conducted according to CPMP/SWP/1042/99 Rev1 with three groups of three male and female NZW rabbits each. Two dose groups of Budesolv (300 µg/mL and 150 µg/mL) and the reference item Rhinocort^®^ aqua 64 were tested. Additionally, reversibility of any occurring effect was assessed in two animals per group and sex at the end of a subsequent 2-week treatment-free period. Animals were clinically evaluated every day for their general condition and for signs of local nasal irritation.

No signs of irritation or edema were observed during the treatment period of 28 days in any of the animals. No clinical symptoms could be detected in any of the animals within the treatment period. No histopathological changes were observed at the application site at the end of the treatment or the recovery period. In conclusion, in comparison to the reference item, treatment with Budesolv revealed no differences regarding local tolerance at the application site.

No toxic effects were observed. No relevant differences were found in body weight development or food and water consumption. The 4-week, twice daily intranasal treatment with the test item (Budesolv 150 µg/mL and 300 µg/mL) or reference item (Rhinocort^®^ Aqua 64) did not reveal any relevant changes of hematology, blood coagulation and clinical biochemistry parameters. Accordingly, no treatment-related effects of both concentrations of budesonide solubilized in Marinosolv^®^ in comparison to the reference item to systemic toxicity were reported.

## 4. Discussion

Highly potent and hydrophobic drugs, such as budesonide, face bioavailability limitations caused by two factors: the low solubility and the low chemical stability in aqueous systems [29]. Due to the low solubility and limitations in tolerability of solubility enhancing components, modern glucocorticoids are generally marketed as suspensions. The present study shows that the platform Marinosolv^®^ is suitable to apply solubilized budesonide in a physiologically tolerable nasal spray formulation. The saponin escin, together with the solvent PG, was able to increase the amount of budesonide in an aqueous solution to more than 400 µg/mL compared to 28 µg/mL water solubility [5,6]. We found that in our system, the concentration of solubilized budesonide can unexpectedly be further increased by the addition of dexpanthenol. It furthermore had the benefit of stabilizing the micellar colloid. The addition of dexpanthenol appears to be essential for the long-term stability of this micelle-based formulation. Typically, dexpanthenol is used in marketed intranasal formulations to improve the tolerability of preparations of decongestions and preservatives by hydrating treated areas and maintaining the mucosal surface integrity [25,30].

Depending on formulation specifics, such as the used solvent, buffer salts, other additives, temperature and pH, a range of CMC values of escin micelles between 0.06 mM (0.00068%) and 2.2 mM (0.025%) has been published [18,31,32,33,34,35,36]. We showed that in the case of Marinosolv^®^ the solubilization effect of escin was based on self-assembling micelle formation due to the fact that the lowest escin concentration effective for solubilization of budesonide coincides with the CMC of escin measured in our system (i.e., 0.01%).

Escin has already been investigated as solubility enhancer for hydrophobic drugs [22] and showed very different solubilization capacities depending on the type of solubilized drug molecule. Vinarov et al., described that a solubilization effect at an escin concentration of 0.5% *w*/*v* was only observed for the steroidal structured investigated drug danazol and not for the large planar aromatic molecule fenofibrate. This phenomenon was explained as a result of ion-dipole interactions between the polar danazol molecules and the charged surfactant head-groups [37]. In contrast to the data published by Vinarov et al., the pre-solvation of budesonide in PG before addition to the micellar formulation is essential in the Marinosolv^®^ system.

The characterization of the hydrodynamic diameter of the escin micelles in Marinosolv^®^ using dynamic light scattering (DLS) was unsuccessful. This is most likely due to the low concentration of escin, the non-spherical shape, and the very heterogenous size distribution of escin micelles. In recent publications, escin micelles formed in solutions were characterized with transmission electron microscopy (TEM). In water at room temperature with unspecified pH, escin micelles formed oblong structures of about 20 nm length that were stacked as larger aggregates [31,33]. Dargel et al. [33], on the other hand, characterized escin micelles in 50 mM phosphate buffer pH 7.4 using TEM as well as small-angle X-ray scattering (SAXS). TEM images revealed mainly rod-like structures (cross-sectional diameter: 25–35 Å, length: 70–150 Å) co-existing with smaller spherical particles. Again, these rod-like structures were made up of smaller discoidal structures. Further experiments using SAXS revealed a temperature-dependent shape. While escin micelles were predominantly oblong at 10 °C, they became ellipsoidal at 40 °C.

Based on previously conducted intranasal local tolerance studies in NZW rabbits and solubility studies, a formulation containing 0.03% escin and 6% dexpanthenol was chosen for further development.

In any intranasally applied glucocorticoid therapy, the goal is to reach a high local tissue concentration and a low systemic exposure of the drug. Due to the mucociliary clearance in the nasal cavity, there is a time restriction for local drug uptake. Permeation needs to occur within 20–30 min after application, otherwise the remaining drug amount is swallowed and can therefore not contribute to the local activity [7,8,38,39]. Consequently, the amount of intranasally applied glucocorticoids that reaches the systemic circulation is the sum of the nasal and oral (swallowed) bioavailable fractions. The contribution of the nasally absorbed fraction of glucocorticoids in suspension is minimal because the majority of the drug is swallowed, and systemic bioavailability will be determined by the absorption from the gastrointestinal tract and the degree of first-pass hepatic inactivation [38]. For budesonide solubilized in Marinosolv^®^, we have demonstrated in permeation kinetics experiments that a significantly higher total drug tissue concentration compared to Rhinocort^®^ aqua 64 is achieved over the whole course of the experiment (Figure 2). This result is even more remarkable as the concentration of budesonide in this formulation is less than a quarter of the budesonide concentration in Rhinocort^®^ aqua 64. Regarding the uptake kinetics of budesonide, Budesolv 300 µg/mL reached a plateau in drug permeation on porcine nasal mucosa in our ex vivo experiment after approximately 15–30 min. Therefore, within the provided short time frame for nasal absorption, with Budesolv 300 µg/mL a high permeated drug content in the tissue is achieved. In addition, our permeation experiments showed that the nasal epithelial tissue is capable of absorbing increasing amounts of budesonide with increasing concentrations of solubilized drug. A formulation with 800 µg/mL budesonide solubilized with the micelle system led to a drug concentration of 150 µg/g budesonide in nasal mucosa after 30 min of incubation.

To test whether the higher ex vivo tissue levels and faster uptake into mucosa could translate into a higher therapeutic effect and a faster onset of action, we performed two in vivo experiments where the inhibition of the inflammatory response to an LPS challenge in the lung of mice by various budesonide formulations was tested. The fast permeation of budesonide in the ex vivo experiments is of utmost importance and a requirement for a potential early onset of therapeutic efficacy in humans. In fact, standard glucocorticoid-containing nasal sprays have to be applied for several days to exert a symptom relief, and it can take up to two weeks to achieve full therapeutic effectiveness [40].

First, a head-to-head comparison of solubilized budesonide (Budesolv 300 µg/mL) with the marketed product Rhinocort^®^ aqua 64 (containing 1.28 mg/mL dispersed budesonide) and placebo in a prophylactic setup was carried out in vivo (Figure 4). At every time point of treatment start (24 h, 18 h and 3 h) before LPS challenge, Budesolv 300 µg/mL resulted in an increased reduction of TNF-α secretion compared to the more concentrated budesonide suspension (Rhinocort^®^ aqua 64). These results indicate that the higher permeation rate found in the ex vivo experiment translated into the in vivo situation, which leads to a stronger anti-inflammatory action of the solubilized glucocorticoid. This further supports the hypothesis that the dispersed formulation, although four times more concentrated, has certain limitations.

A second in vivo experiment was performed to mimic a therapeutic set-up by treating mice 15 min after LPS instillation (Figure 5). In this experiment, Budesolv 300 µg/mL resulted in a significant higher reduction in TNF-α secretion than Rhinocort^®^ aqua 64, which did not show any effect relative to placebo in this setting. Thus, we conclude that 15 min was not enough time for budesonide from the suspension to dissolve and permeate into the tissue in physiologically active concentrations. This further supports the hypothesis that the concentration of solubilized and therefore freely permeable budesonide rather than the total applied dose of the drug is a key parameter for anti-inflammatory effectivity.

As β-CDs were already investigated to re-formulate budesonide [12,14], a formulation containing 300 µg/mL budesonide complexed by using randomly methylated β-CD was included in our ex vivo and in vivo experiments. This type of β-CD is already used for a 17β-Estradiol nasal spray (Aerodiol, Les Laboratoires Servier) and was therefore considered as an adequate comparator system. In the ex vivo experiment, 300 µg/mL budesonide complexed with β-CD permeated into nasal mucosa to similar tissue levels achieved with Rhinocort^®^ aqua 64. Published in vitro experiments showed that the complexation of budesonide with HP-β-CD led to enhanced drug solubility and release rate compared to a suspension [41]. However, after in vivo lung application, the budesonide permeability in the presence of HP-β-CD complexes was not increased [10]. The authors observed a similar in vivo anti-inflammatory effect after treatment with 2.5 times lower budesonide concentrations complexed with HP-β-CD compared to a dispersed budesonide formulation (non-complexed formulation). Therefore, the authors concluded that the drug permeation into tissue is related to the free budesonide fraction rather than the complexed fraction. They further concluded that the CD complexing of budesonide leads to a slow release formulation. In our therapeutic in vivo experiment in mice, both Rhinocort^®^ aqua 64 and 300 µg/mL budesonide complexed with β-CD failed to significantly reduce the inflammation induced by LPS compared to placebo. In two environmental challenge chamber studies with allergic subjects the therapeutic equivalence of β-CD (Captisol)-complexed budesonide (640 µg/mL) nasal spray and the marketed Rhinocort^®^ aqua 32 (640 µg/mL dispersed budesonide) nasal spray, was tested. In a pooled analysis, equivalence of solubilized and dispersed budesonide in the same concentration was demonstrated [11], but the studies failed to demonstrate an early onset of action.

We acknowledge that our animal model is not directly disease relevant in mimicking allergic rhinitis or asthma. However, it is well established that corticosteroids such as budesonide inhibit both, inflammation due to an allergic reaction and an inflammation due to bacterial infection (mimicked by LPS application). TNF-α is a hallmark cytokine in both diseases, allergy and bacterial infection, and thus a relevant readout to determine the efficacy of the anti-inflammatory drug budesonide. Therefore, a mechanistic setup with considerably less temporal involvement on the animals’ side was chosen. The data obtained from the LPS challenge model therefore serve as an indirect measurement for budesonide tissue permeation with a subsequent biological effect.

From a product development perspective, a remedy with budesonide being present in a particle free formulation has several additional advantages. Such a product can be sterilized by terminal sterile filtration, while suspension products need to rely on preservatives to maintain the microbiological quality required for nasal products. As there are no particles that can settle during periods where the product is at rest, there is no need for shaking the product vial prior to application to ensure a homogeneous distribution of the drug in the product and dose uniformity throughout container life. Such a product can be manufactured using a standard manufacturing process that can easily be upscaled and produced on industrial scale.

In conclusion, we have designed an escin-based micellar formulation of budesonide applicable for intranasal use. The components, especially the surfactant escin, are used in pharmacologically/physiologically acceptable concentrations as shown in a 4-week GLP local tolerance study of intranasally applied Budesolv. In ex vivo experiments, we showed faster permeation and significantly higher mucosal concentrations of budesonide when applied in solubilized form as Budesolv. In a murine pulmonary inflammation model, we showed that 300 µg/mL Budesolv had a stronger anti-inflammatory effect in a prophylactic treatment set-up, as well as in a therapeutic treatment setting than the marketed budesonide product.

Therefore, we conclude that the application of solubilized budesonide results in a faster onset of action and that the good tissue permeation characteristics offer the opportunity to reduce the concentration of applied drug without the loss of effectivity and subsequently reducing systemic side effects.

## 5. Patents

The authors Andreas Grassauer, Eva Prieschl-Grassauer, Martina Morokutti-Kurz, Cornelia Siegl, and Sabine Nakowitsch are inventors of the patent #WO2017009480A1, “Method for improving aqueous solubility of water-insoluble or slightly water-soluble drugs”. The patent is held by Marinomed Biotech AG and is related to the content of the manuscript.

## Figures and Tables

**Table 1:** Results of the statistical analysis of the permeation of budesonide into porcine nasal mucosa after incubation with different formulations.

Formulations	15 min	30 min	45 min	60 min
Budesolv 300 µg/mL vs. Rhinocort^®^ aqua 64	*p* = 0.005 **	*p* = 0.002 **	*p* = 0.002 **	*p* = 0.003 **
β-CD 300 µg/mL vs. Rhinocort^®^ aqua 64	*p* = 0.086	*p* = 0.021 *	*p* = 0.276	*p* = 0.492
Budesolv 300 µg/mL vs. β-CD 300 µg/ml	*p* = 0.001 **	*p* = 0.010 *	*p* = 0.007 **	*p* = 0.001 **

Statistical analysis was done using paired Student’s *t*-test, ** *p* < 0.01 and * *p* < 0.05.
